# 0100. Apelin is cardioprotective and life-saving over dobutamine in a murine model of endotoxin-induced myocardial dysfunction

**DOI:** 10.1186/2197-425X-2-S1-P11

**Published:** 2014-09-26

**Authors:** O Lesur, F Chagnon, A Murza, P Sarret, E Marsault, D Salvail

**Affiliations:** University de Sherbrooke, ICU / Medicine / CHUS, Sherbrooke, Canada; ICU/Medicine/CRCHUS, Sherbrooke, Canada; IPS/ U de Sherbrooke, Pharmacology, Sherbrooke, Canada; IPS/ U de Sherbrooke, Physiologie, Sherbrooke, Canada; IPS Therapeutique Inc, Sherbrooke, Canada

## Introduction

Dobutamine (DOB) is the actual recommended b-adrenergic inotropic drug to support sepsis-induced myocardial dysfunction when cardiac output index is still low after preload correction. In this context, DOB cardiovascular response predicts outcome in septic shock. Alternative supportive and safer therapies are however mandatory because:

1. only 35-45% of septic patients do respond to DOB, and
2. numerous side-effects of DOB can be observed, including potential harmful impact on cardiomyocyte function. Apelin (APLN) is a powerful inotrope, is widely expressed by the cardiovascular system with its receptor APJ-R, and should be considered as an alternative noncatecholaminergic support.

## Objectives

Perform a comparative evaluation of APLN-13 (APLN active peptide) vs DOB in terms of hemodynamic efficacy, cardioprotection and outcome in a model of “sepsis-induced cardiac dysfunction”.

## Methods

A rat model of LPS-induced myocardial dysfunction (*E. Coli* 055:B5, 10-12mg/kg intraperitoneal).

### Interventions

APLN-13 vs DOB (0.23 vs 7.5µg/kg/min i.v continuous infusion respectively, as determined by preliminary experiments with or without “in parallel” fluid resuscitation (saline, 2mL/kg/hr).

### Time-course

0-6-18h.

### Outcomes

*in vivo*: echocardiography, final hemodynamics; urine output, weight and plasma volume variation, survival study, *ex vivo*: Langendorff dP/dt, *in vitro*: APJ-R and beta1-adrenergic receptor (AR) myocardial expressions, Pi3K/Akt/GSK3/mTOR activation-expression profiles, cTnI (troponin, myocardial injury) and cleaved caspase-3 (apoptosis).

## Results

Both drugs restored LPS-induced fall of left ventricular ejection fraction (LVEF) with dominant chronotropic impact and mean arterial pressure (MAP) restoration for DOB, and lower peripheral vascular resistances (PVR) for APLN. APJ-R but not beta1 AR myocardial expressions were upregulated by LPS challenge (p< 0.05). The deepness the induced LVEF drop, the higher the dP/dt response to APLN but not to DOB (p< 0.05). In 18h LPS-challenged hearts, Langendorff assays peak dP/dt responses were 3nM and 100nM for APLN and DOB, respectively (p< 0.05). Combining fluid resuscitation with APLN infusion declined urine output (UO) with plasma volume (PV) expansion, whereas DOB induced less PV expansion but more UO (p< 0.05). Survival proportions were clearly distinctive (p< 0.05).

**Figure 1:**
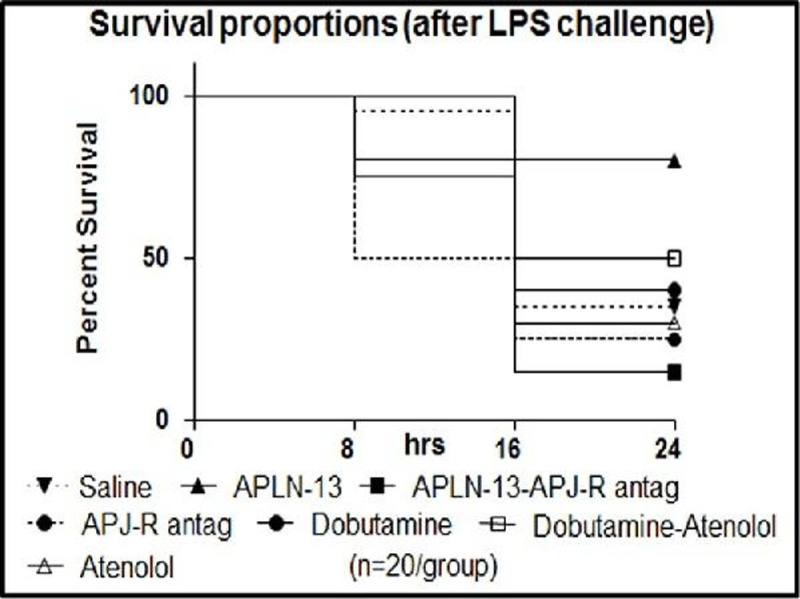
APLN survival

APLN further dampened down LPS-induced overphosphorylation/inhibition of Pi3K/Akt/mTOR/GSK3 and reduced injury/apoptosis (i.e. cTNI and cleaved caspase-3 expressions).

## Conclusions

APLN potentially offers distinctive mechanisms of hemodynamics, cardioprotective effects, and survival benefits, over DOB. Chemical optimization of APLN-13 with more extensive preclinical data, would pave the way for first phase clinical trials.

